# Adherence to the Mediterranean Diet and Perceived Immunity Among the Saudi Population: A Cross-Sectional Study

**DOI:** 10.7759/cureus.34963

**Published:** 2023-02-14

**Authors:** Hany K Mostafa, Ayat T El-Zayat, Osamah Abdullah A Alraddadi, Mohanad Abulaban

**Affiliations:** 1 Histology and Cell Biology, Fakeeh College for Medical Sciences, Jeddah, SAU; 2 Medical Science, Ain Shams University, Cairo, EGY; 3 Clinical Sciences, Fakeeh College for Medical Sciences, Jeddah, SAU

**Keywords:** nutritional behavior, saudi population, general health, immunity, mediterranean diet

## Abstract

Background

The Mediterranean diet (MedDiet) is a model of a sustainable dietary pattern. It has a protective role against coronary heart diseases, but nowadays it is hypothesized against many other diseases.

Aim of the study

This study aims to assess the prevalence of adherence to the Mediterranean diet and its association with immune status among the general Saudi population.

Subjects and methods

A cross-sectional study design was conducted over a sample size of 327 Saudi adults living in Jeddah. A self-administered online questionnaire was distributed in a digital Google Form via various social networks using the convenient sampling technique. The questionnaire had four major divisions: sociodemographic, general health characteristics, adherence to MedDiet, and immune status. The data were collected, validated, and subjected to statistical analysis.

Results

The study showed a statistically significant association between adherence to MedDiet and perceived immune status (p-value <0.05). However, the associations between adherence to MedDiet and having any chronic illness, and perceived general health were not statistically significant (p-value >0.05). Multivariable regression models showed that sex and occupation are significant predictors of adherence to MedDiet.

Conclusion

This study concluded that despite low adherence to MedDiet among Saudis, there was an association between adherence to MedDiet and immune status. This study recommends the implementation of MedDiet as an intervention for the management of chronic diseases to be considered by policymakers and guideline developers.

## Introduction

The Mediterranean diet is a way of eating that's based on the traditional cuisines of Greece, Italy, and other countries that border the Mediterranean Sea. The traditional Mediterranean diet is characterized by high consumption of vegetables, fruits, nuts, legumes, and unprocessed cereals; low consumption of meat and meat products; and moderate daily dairy consumption (with the exception of long-preserved cheeses) [1].

The Mediterranean diet is one of the most popular worldwide dietary styles due to its collection of foods rich in antioxidants and anti-inflammatory nutrients [2]. Many studies have agreed that there is a strong and inverse relationship between a high level of Mediterranean diet adherence and immunity [3]. The Dietary Guidelines for Americans (Dietary Guidelines) 2020-2025 recommended that Mediterranean diet components be consumed in order to prevent and reduce morbidity from cardiovascular disease, which is the third leading cause of death worldwide [4]. Obesity and metabolic syndromes were found to be less likely to develop in adults who followed the Mediterranean diet [5].

The Mediterranean diet plays a significant role in cancer prevention and reduces the risk of many types of cancer, such as breast, prostate, and lung cancer. A high fiber intake and a moderate milk intake help reduce the incidence of various types of cancer. Cooking red meat at high temperatures may increase the risk of developing cancers such as colorectal cancer, stomach cancer, and prostate cancer [6].

Beneficial roles of the Mediterranean diet also appear in chronic degenerative diseases. Previous studies showed that chronic diseases such as diabetes mellitus, hypertension, and thromboembolic diseases, which are rising in prevalence among the general population, were better controlled by the Mediterranean diet [2,7].

Nutrients, including macro- and micronutrients, vitamins, and minerals, play a functional role throughout the immune response to diseases, and a lack of any could weaken immunity. Diet provides human beings with different nutritional requirements; thus, the pattern of an individual's diet can affect their immune status in either a positive or negative way [3].

The WHO also recognizes that diet plays an important role in preventing non-infectious disease, and unhealthy food lifestyles as well as other adverse lifestyle health behaviors are recognized as prime factors [8]. The Mediterranean diet is composed mainly of foods that best provide the micronutrients required for immune function as well as the appropriate amounts of macronutrients that enhance this function [1].

According to the Saudi Health Interview Survey, only 5.2% of 10,735 Saudis consumed fruits, 7.5% consumed vegetables, 31.4% consumed nuts, and 44.7% consumed fish [9]. However, previous research has reported a low level of adherence to the Mediterranean diet among non-diabetic Saudi patients at 25.7%. [10] For the Gulf area, the Saudi population had significantly lower adherence to MedDiet compared to the Omani and Kuwaiti populations [11].

Ultimately, the research in the last few years has been focused on adherence to the Mediterranean diet and its relation to the health and immune status of the population. To the best of our knowledge, this study is the first to assess the adherence to the Mediterranean diet among a sample of the Saudi Arabian population and its association with their perceived general health and perceived immune status using a cross-sectional study.

## Materials and methods

Study design, sample size determination, and sampling procedure

A cross-sectional study design was conducted over a sample of 327 Saudi adults after calculating the sample size at a 95% confidence level and an alpha error of 5% using the Epi-Info by Centers for Disease Control (CDC) software calculator based on the percentage of adequate adherence to the Mediterranean diet among the Saudi population (25.7%) [10]. The calculated sample size was 294 participants, but the study included 327 participants. The participants were enrolled by convenient sampling via an online questionnaire, for which the link was distributed, and those who responded first were the sample (Appendix 1).

Study population and eligibility criteria

Inclusion criteria were: adults over the age of 18 who could give consent, were of both genders, were Saudi citizens, could read and write, and had access to the internet to fill out the online form. We excluded any healthcare providers or medical students because their nutritional knowledge may affect their practices or the general population who follow a specific diet, such as the Mediterranean diet. 

Data collection and study variables

A self-administrated online questionnaire over the duration of May 2021 to July 2021 was distributed in a digital Google Form via different social networks, including Facebook, Twitter, WhatsApp, and others. The estimated time to fulfill the questionnaire was about 10-12 minutes. The first part of the questionnaire included a brief introduction explaining the aim of the study, volunteer participation, a declaration of confidentiality and anonymity of the data, and also informed consent to participate in the study. The participants had the right to refuse participation or to withdraw without any reasons or negative consequences. Also, the authors' contact information was available for the participants to provide any needed clarification.

The questionnaire included four sections, as follows:

A. Sociodemographic data of the studied population: age, sex, education, marital status, residence, and occupation.

B. General health characteristics of the studied population: the presence of chronic illness, the administration of immunosuppressive medications, the competency to follow health-care provider instructions, follow-up, and treatment for becoming ill; one-item perceived general health on a scale of 0 (very poor) to 10 (excellent); and one-item perceived immune status on a scale of 1 (very poor) to 10 (excellent) [12].

C. The Mediterranean Diet Adherence Screener (MEDAS) is a 14-item validated questionnaire used to assess participants' adherence to the Mediterranean diet (MedDiet). These items asked about the consumption of the following categories: olive oil, vegetables, fruit, meat, butter, margarine, or cream; sweet or carbonated beverages; wine; legumes; fish or shellfish; commercial sweets or pastries (not homemade); nuts (including peanuts); and chicken, turkey, or rabbit meat. Each of the 14 items is scored one or zero, depending on whether participants adhere to each MedDiet component or not. The MEDAS items and the criteria for scoring one point are: If these conditions were not met, an item was assigned a score of 0. The resulting MEDAS-derived MedDiet score ranged from zero to 14. The cutoff for adherence was defined as ≥ eight points [13].

D. Validated a seven-item Immune Status Questionnaire (ISQ) to assess immune functioning. Each item of the ISQ was scored as follows: Zero = Never, one = Sometimes, two = Regularly, three = Often, and four = Always (almost). To obtain the final ISQ score, translate the "raw" ISQ scores as follows: Interpretation: zero = very poor, 10 = excellent perceived immune status (Table 1). The cut-off for reduced immune functioning: ISQ < six [12].

**Table 1:** Immune Status Questionnaire (ISQ) scoring instruction (12)

Immune Status Questionnaire (ISQ) scoring	
Raw score	Final score
≥15	0
14	1
13	2
11, 12	3
10	4
8, 9	5
7	6
6	7
5	8
3, 4	9
≤2	10

Validity and reliability

A forward-backward technique was used for translating the questionnaires. The authors translated the questionnaires from English to Arabic first. An independent, proficient English speaker did a translation from Arabic to English, followed by a second round of translation from English to Arabic. A certified English translator compared the original and back-translated versions of the English questionnaire, while a medical professor compared two translated Arabic versions. All discrepancies were resolved, and the final Arabic versions of both questionnaires were matched.

The questionnaire was then evaluated for face validity through a pilot study in which it was administered to 30 participants to ensure the clarity and simplicity of the questions and answer choices. Feedback from the pilot study was followed by minor modifications to a few questions. These questions were about the general characteristics of health. After that, the questionnaire was distributed for data collection.

Data management and statistical analysis

The data were coded, entered, and manipulated using Microsoft Excel (2019 version). Data analyses were performed using IBM Statistical Package for the Social Sciences (SPSS) software (version 20) [14]. Socio-demographic characteristics, general health characteristics, MedDiet adherence, and immune functioning status were described using descriptive statistics, including frequencies and percentages (%). The scores for the 1-item perceived general health and 1-item perceived immune status were described using descriptive statistics of mean, standard deviation (SD), median, and range (minimum-maximum).

Chi-square and Fisher’s exact tests were used for testing the statistical significance of categorical data. Fisher's exact test was used whenever the Chi-square test assumptions were violated (i.e., when more than 20% of the expected values were less than five or any of the cell values equaled 0). Comparisons between 1-item perceived general health and 1-item perceived immune status among adherent and non-adherent groups were assessed for significance using the Mann-Whitney test. Multivariable regression analysis was used to assess predictors of adherence to MedDiet. A p-value < 0.05 was considered statistically significant based on the level of confidence of 95%. The study was ethically approved by the IRB of Fakeeh College of Medical Sciences under approval no. 149/IRB/2020.

## Results

A total of 327 participants were studied. The majority of the participants were within the age range of 18-30 years (n=270, 82.6%). Most of them were women (n=270, 82.6%). Most had college-level education (n=240, 73.4%); and were single (n=262, 80.1%). The participants resided in Jeddah (n=167, 51.1%) and Makkah (n=108, 33%); and most were students (n=205, 62.7%) (Table 2).

**Table 2:** Sociodemographic data of the studied population.

Sociodemographic data		Frequency	Percent
Age	18-29	270	82.6
	30-39	34	10.4
	40-49	10	3.1
	50-59	7	2.1
	60-70	6	1.8
Sex	Male	57	17.4
	Female	270	82.6
Education	Primary	1	0.3
	Preparatory	20	0.5
	High school	76	23.2
	College	240	73.4
	Post-graduate	8	2.4
Marital status	Single	262	80.1
	Married	60	18.3
	Divorced	5	1.5
Residence	Jeddah	167	51.1
	Makkah	108	33
	Riyadh	12	3.7
	Madinah	17	5.2
	Other	23	7
Occupation	Student	205	62.7
	Office employee	30	9.2
	Field employee	22	6.7
	Other	23	7
	Not working	40	12.2
	Retired	7	2.1

A few of the participants complained of chronic illness, including diabetes mellitus, hypertension, cardiac, renal, or hepatic disease (n=28, 8.6%); only nine (n=9, 2.8%) were on immunosuppressive medications (any medication that the participant was informed by the physician caused immunosuppression); and a few were compliant with the instructions of their health-care provider, follow-up, and treatment after getting sick (n=21, 6.4%). The perceived general health score among the studied participants was 7.2±1.9 while the perceived immune status was 7.4±2.1 (Table 3). The perceived immune status was then classified into low status (n=184, 56.3%) and high status (n= 143, 43.7%) (Table 4).

**Table 3:** General health characteristics of the studied population.

General health characteristics		Frequency	Percent
Chronic illness	Present	28	8.6
	Absent	299	91.4
Immunosuppressive medications	Yes	9	2.8
	No	318	97.2
Competency to follow-up and treatment upon getting ill	Competent	21	6.4
	Non-competent	47	14.4
	Not sure	259	79.2
Perceived general health	Mean ±SD	7.2±1.9	
	Median (min-max)	8(1-10)	
Perceived immune status	Mean ±SD	7.4±2.1	
	Median (min-max)	8(1-10)	

**Table 4:** Association between adherence to the Mediterranean diet and immune status. *Chi-square, statistically significant; Frequency (%)

Adherence	Immunity		Total	p-value
	Low	High		
Non-adherent	175 (95.1)	119(83.2)	294 (89.9)	0.0001*
Adherent	9 (4.9)	24 (16.8)	33 (10.1)	
Total	184 (56.3)	143 (43.7)	327 (100)	

Despite the low prevalence of adherence to the MedDiet (n=33, 10.1%), the association between adherence to the Mediterranean diet and perceived immune status was statistically significant (p-value < 0.05) (Table 4). There was no statistically significant link between having a chronic illness, taking immunosuppressive medications, and being able to follow up and treat oneself after becoming ill. However, the relationship between Mediterranean diet adherence and perceived immune status was statistically significant (P-value < 0.05) (Table 5).

**Table 5:** The association between adherence to the Mediterranean diet and general health characteristics. *Mann-Whitney, statistically significant.

General health characteristics		Adherence		p-value
		Non-adherent	Adherent	
Chronic illness frequency (%)	Present	27(9.2)	1(3)	0.335
	Absent	267(90.8)	32(97)	
Immunosuppressive medications frequency (%)	Yes	9(3.1)	0(0)	0.606
	No	285(96.9)	33(100)	
Competency to follow-up and treatment for getting ill frequently (%)	Competent	20(6.8)	1(3)	0.064
	Non-competent	228(77.6)	1(3)	
	Not sure	46(15.6)	31(93.9)	
Perceived general health	Mean ±SD	7.17±1.9	7.7±1.9	0.119
	Median (min-max)	7.5 (1-10)	8(4-10)	
Perceived immune status	Mean ±SD	7.3±2	8.2±2	0.002*
	Median (min-max)	8(1-10)	9(1-10)	

As regards the multivariable regression models, only the male gender increased the probability of adherence to MedDiet by 2.285 (β = 2.285, 95% confidence interval (CI): 1.021, 5.112, p = 0.044), occupation (those retired or not working) decreased the probability of adherence to MedDiet by 0.9 (β = 0.9, 95% CI: 0.892, 1.376, p = 0.001) and perceived immune status (where those reporting their immune status as good) increased the probability of adherence to MedDiet by 1.302 (β = 1.302, 95% CI: 1.053, 1.608, p = 0.015), and these three variables were statistically significant predictors for the adherence of Mediterranean diet (Table 6).

**Table 6:** Multivariable regression analysis of predictors of adherence to the Mediterranean diet. *Statistically significant.

Independent variables	B	p-value	Exp(B)	95% C.I. for EXP(B)	
				Lower	Upper
Age	0.052	0.814	1.053	0.682	1.627
Sex	0.826	0.044*	2.285	1.021	5.112
Education	-1.477	0.179	0.566	0.247	1.297
Marital status	-0.11	0.979	0.989	0.44	2.223
Residence	0.219	0.112	1.245	0.951	1.63
Occupation	0.103	0.001*	0.9	0.892	1.376
Chronic illness	1.174	0.257	3.236	0.425	24.622
Immunosuppressive medications	0.104	0.31	0.056	-0.097	0.304
Competency to follow-up and treatment upon getting ill	0.981	0.072	2.668	0.915	7.781
Perceived general health	0.161	0.129	1.175	0.954	1.446
Perceived immune status	0.246	0.015*	1.302	1.053	1.608

## Discussion

The Mediterranean diet was inscribed, in November 2010, on the Representative List of the Intangible Cultural Heritage of UNESCO. The nomination was supported by Italy, Spain, Greece, and Morocco. Cyprus, Croatia, and Portugal joined in 2013 [15]. The MedDiet was first presented by Ancel Keys in the 1960s [16]. The MedDiet includes high consumption of vegetables, fruits, nuts, legumes, and unprocessed cereals; low consumption of meat and meat products; and moderate daily dairy consumption (with the exception of long-preserved cheeses) [16].

This study examined the adherence to MedDiet among a sample of 327 Saudis, and it showed low adherence (n= 33, 10.1%). According to the Saudi Health Interview Survey (SHIS), which was a national multistage survey of individuals aged 15 years or older performed between April and June 2013 and which was the first study to describe dietary patterns among Saudi adults, dietary guideline recommendations were met by only 5.2% of individuals for fruits, 7.5% for vegetables, 31.4% for nuts, and 44.7% for fish. On the other hand, the consumption of processed foods and sugar-sweetened beverages was high among the Saudi population [17]. Their low adherence to fruits, vegetables, nuts, and fish is consistent with the observed low adherence to MedDiet, which includes similar items among the studied Saudi population.

The observed low adherence to MedDiet can be explained by many factors. First, there is low awareness of MedDiet and its potential benefits due to a lack of awareness programs, according to some authors [13]. Second, MedDiet's inclusion of alcohol consumption is a repulsive factor for Muslims [18]. Third, the study was conducted in a non-Mediterranean country, Saudi Arabia, where fish, for example, is not a main dish for this population.

This study revealed that adherence to MedDiet is associated with a high perceived immune status. The MedDiet has been linked to significant increases in many risk factors, including oxidative stress and inflammation, according to the literature [19]. Evidence from many clinical trials has implied that the MedDiet reduces vascular inflammation, oxidative stress, and endothelial on many biomarkers such as interleukin-18 (IL-18), matrix metalloproteinase (MMP)-9 or transforming growth factor-beta (TGFβ) [20]. Moreover, the high content of antioxidants in MedDiet, such as vitamin C, beta-carotene, and polyphenols, exerts immunomodulatory and anti-inflammatory effects, reducing the risk of many diseases. [21] It was also found to have an effect on the upregulation of the circulating cluster of differentiation 40 (CD40)+CD86+ cells for the initiation of an adaptive immune response and T-cell activation [22]. This explains the positive effect of MedDiet on immune status.

Previous studies all agreed on this finding. A one-year consumption of a Mediterranean-like dietary pattern study in the United Kingdom showed that adherence to an individually tailored Mediterranean-like dietary pattern for one year modified a large variety of parameters of immune function in healthy, elderly subjects. [22] Casas et al., in their review, provided evidence of MedDiet's effectiveness as a protective agent against several diseases [20]. Farajian et al. studied 4,786 children in Greece and came to the conclusion that adherence to MedDiet lowers the risk of developing obesity [23]. Menotti et al. went along with our study when they confirmed adherence to MedDiet and a low incidence of coronary heart disease in 1,139 men and women in Italy [24].

The current study showed that being male increases the probability of adherence to MedDiet. Leblanc et al. studied the gender differences in the long-term effects of a nutritional intervention program promoting MedDiet. Men reported larger decreases in consumption of red and processed meat and larger increases in consumption of whole fruit intakes than women, which is consistent with the components of MedDiet [25]. However, Novak et al. found that low adherence to a Mediterranean diet was associated with being female [26]. On the other hand, Romaguera et al. and Trichopoulou et al. found no significant relationship between adherence to a MedDiet and obesity in both genders in different studies in European countries and Cyprus, respectively [27,28]. This difference could be explained by theories about the association of a good diet with masculinity among Saudi Arabian males.

In this study, another independent predictor of adherence to MedDiet was the occupation, as being retired or not working decreased the likelihood of adherence to MedDiet. This could be explained by the fact that adherence to any dietary guideline depends on the socioeconomic status of the population because of the relatively high cost of foods that provide the required items of the MedDiet [29]. According to Álvarez et al., people who had a higher education, a better occupation, and were older had a stronger affinity for MedDiet [30]. 

Limitations of the study

The first limitation of this study is the cross-sectional study design, as it assesses exposure and outcome at the same point in time without proving the temporal relationship. This may limit its ability to draw valid conclusions from the association between them. Another limitation is the non-random sampling technique used in the online survey; the sample may not be fully representative of the Saudi population. This sampling technique may undermine the generalization of the findings from the studied sample to the whole Saudi population. As the respondents selected themselves for the sample, the volunteer bias was a potential risk that makes the population a bit different from the entire population.

Recommendations

Based on the findings of the current study, more interventional programs should be held to raise awareness among Saudis about the components of MedDiet and its impact on health and immune status. The implementation of MedDiet as an intervention for the management of chronic diseases should be considered by policymakers and guideline developers. Further research should be done using a large sample size from many different cities and utilizing interviewing methods by a well-trained interviewer to get more conclusive results.

## Conclusions

In this work, we found that there was a low adherence to MedDiet among the Saudi population. However, there was a statistically significant association between adherence to MedDiet and a good perceived immune status. The gender and occupation of the Saudi population were significant predictors of adherence to MedDiet.

## Appendices

Appendix 1

Questions about sociodemographic data

1. What is your age (in years)?

2. What is your gender? Male- Female

3. What is your highest level of education? Primary, Preparatory, High School, College, and Post-Graduate

4. What is your marital status? Single, married or divorced

5. Where do you live? Jeddah, Makkah, Riyadh, Madinah, and Other

6. What is your current occupation? Student; office worker; field worker; unemployed; retired

Questions about general health characteristics:

1. Do you have a chronic illness like diabetes, hypertension, cardiac, renal, or hepatic disease? Yes/No

2. Do you receive any immunosuppressive medications (any medication about which the participant was informed by the physician that it causes immunosuppression)? Yes/No

3. Your level of competence (adherence) to scheduled follow-up and treatment when you become ill: competent, non-competent, not sure

4. Describe how you perceive your general health on a scale of 0 to 10 (0= very bad, 10= very good).

5. Describe how you perceive your immune status on a scale of 0 to 10 (0= very bad, 10= very good).

Questions about adherence to the Mediterranean diet:

**Table 7:** Validated 14-item Questionnaire of Mediterranean diet adherence. [13]

1. Do you use olive oil as the main culinary fat?	Yes
2. How much olive oil do you consume in a given day (including the oil used for frying, salads, out-of-house meals, etc.)?	≥4 tbsp
3. How many vegetable servings do you consume per day? (1 serving: 200 g (side dishes count as half a serving))	≥2 (≥1 portion raw or as a salad)
4. How many fruit units (including natural fruit juices) do you consume per day?	≥3
5. How many servings of red meat, hamburger, or meat products (ham, sausage, etc.) do you consume per day? (1 serving: 100–150 g)	<1
6. How many servings of butter, margarine, or cream do you consume per day? (1 serving: 12 g)	<1
7. How many sweet or carbonated beverages do you drink per day?	<1
8. How much wine do you drink per week?	≥7 glasses
9. How many servings of legumes do you consume per week? (1 serving: 150 g)	≥3
10. How many servings of fish or shellfish do you consume per week? (1 serving: 100–150 g of fish, 4–5 units, or 200 g of shellfish)	≥3
11. How many times per week do you consume commercial sweets or pastries (not homemade), such as cakes, cookies, biscuits, or custard?	<3
12. How many servings of nuts (including peanuts) do you consume per week? (1 serving 30 g)	≥3
13. Do you preferentially consume chicken, turkey, or rabbit meat instead of veal, pork, hamburger, or sausage?	Yes
14. How many times per week do you consume vegetables, pasta, rice, or other dishes seasoned with sofrito (sauce made with tomato and onion, leek, or garlic and simmered with olive oil)?	≥2

Questions about perceived immune status

Please indicate how often you have had the following complaints in the past 12 months:

**Table 8:** Immune Status Questionnaire (ISQ) [12]

	Never	Sometimes	Regularly	Often	Almost (always)
Sudden high fever					
Diarrhea					
Headache					
Skin problems (e.g. acne & eczema)					
Muscle and joint pain					
Common cold					
Coughing					
